# Outcome-Orientated Organ Allocation—A Composite Risk Model for Pancreas Graft Evaluation and Acceptance

**DOI:** 10.3390/jcm13175177

**Published:** 2024-08-31

**Authors:** Sophie Reichelt, Robert Öllinger, Fabian Halleck, Andreas Kahl, Nathanael Raschzok, Axel Winter, Max Magnus Maurer, Lukas Johannes Lehner, Johann Pratschke, Brigitta Globke

**Affiliations:** 1 Department of Surgery, University Hospital of Bonn, Venusberg-Campus 1, 53127 Bonn, Germany; sophie.reichelt@ukbonn.de; 2 Department of Surgery CCM|CVK, Charité—Universitätsmedizin Berlin, Corporate Member of Freie Universität Berlin and Humboldt Universität zu Berlin, Augustenburger Platz 1, 13353 Berlin, Germany; robert.oellinger@charite.de (R.Ö.); nathanael.raschzok@charite.de (N.R.); axel.winter@charite.de (A.W.); max-magnus.maurer@charite.de (M.M.M.); johann.pratschke@charite.de (J.P.); 3 Department of Nephrology and Medical Intensive Care CCM|CVK, Charité—Universitätsmedizin Berlin, Corporate Member of Freie Universität Berlin and Humboldt Universität zu Berlin, Augustenburger Platz 1, 13353 Berlin, Germany; fabian.halleck@charite.de (F.H.); andreas.kahl@charite.de (A.K.); 4 Berlin Institute of Health at Charité—Universitätsmedizin Berlin, BIH Academy, Clinician Scientist Program, Charitéplatz 1, 10117 Berlin, Germany; 5 Department of Radiology CCM|CVK, Charité—Universitätsmedizin Berlin, Corporate Member of Freie Universität Berlin and Humboldt Universität zu Berlin, Augustenburger Platz 1, 13353 Berlin, Germany; lukas.lehner@charite.de

**Keywords:** pancreas transplantation, early graft loss, risk score

## Abstract

**Background**: Pancreas transplantation (PTX) remains the most effective treatment to prevent long-term complications and provide consistent euglycemia in patients with endocrine pancreatic insufficiency, mainly in type I diabetic patients. Considering early graft loss (EGL) and the perioperative complication rate, an optimal risk stratification based on donor risk factors is paramount. **Methods**: In our single-center study, we retrospectively assessed the risk factors for EGL and reduced graft survival in 97 PTXs (82 simultaneous pancreas and kidney [SPK], 11 pancreases transplanted after kidney [PAK] and 4 pancreases transplanted alone [PTA]) between 2010 and 2021. By statistically analyzing the incorporation of different donor risk factors using the Kaplan–Meier method and a log-rank test, we introduced a composite risk model for the evaluation of offered pancreas grafts. **Results**: The overall EGL rate was 6.5%. In the univariate analysis of donor characteristics, age > 45 years, BMI > 25 kg/m^2^, lipase > 60 U/L, cerebrovascular accident (CVA) as the cause of death, mechanical cardiopulmonary resuscitation (mCPR), cold ischemia time (CIT) > 600 min and retrieval by another center were identified as potential risk factors; however, they lacked statistical significance. In a multivariate model, age > 45 years (HR 2.05, *p* = 0.355), BMI > 25 kg/m^2^ (HR 3.18, *p* = 0.051), lipase > 60 U/L (HR 2.32, *p* = 0.148), mCPR (HR 8.62, *p* < 0.0001) and CIT > 600 min (HR 1.89, *p* = 0.142) had the greatest impact on pancreas graft survival. We subsumed these factors in a composite risk model. The combination of three risk factors increased the rate of EGL significantly (*p* = 0.003). Comparing the pancreas graft survival curves for ≥3 risk factors to <3 risk factors in a Kaplan–Meier model revealed significant inferiority in the pancreas graft survival rate (*p* = 0.029). **Conclusions**: When evaluating a potential donor organ, grafts with a combination of three or more risk factors should only be accepted after careful consideration to reduce the risk of EGL and to significantly improve outcomes after PTX.

## 1. Introduction

Pancreas transplantation (PTX), in different combinations, is highly effective in achieving euglycemia, but the procedure is prone to complications associated with the donor organ. Pancreas transplantation alone (PTA) is indicated for insulin-dependent diabetes with life-threatening hypoglycemic unawareness (HU) and high risk of secondary complications [1]. Simultaneous pancreas–kidney transplantation (SPK) remains the gold standard for type 1 diabetes with end-stage renal disease (ESRD) [2,3].

Type 1 diabetes leads to kidney damage up to ESRD, accelerates atherosclerosis and causes neuropathy and retinopathy, accompanied by decreased quality of life (QOL) and life expectancy [2]. PTX is an effective treatment for insulin-dependent diabetes with or without kidney dysfunction, enables permanent euglycemia and prevents the progression of secondary complications [1,4,5]. All types of PTX, including SPK, pancreas after kidney transplantation (PAK) and PTA, can increase long-term patient survival and offer an improvement in QOL [1,5]. In their 2020 annual data report, the Organ Procurement and Transplantation Network reported a 1-year graft survival of 93% for SPK and PAK, and 90% for PTA [6].

With an incidence of about 10%, early graft loss (EGL) continues to be a common complication [7,8]. The most frequent etiology for EGL is vascular graft thrombosis, occurring in 3 to 10% of cases [9], followed by graft pancreatitis, graft necrosis, fistula and bleeding [7,9,10]. EGL in SPK is associated with an increased risk of kidney graft failure and reduced patient survival [8]. These outcome parameters can be influenced by graft-related parameters, as well as perioperative parameters such as immunosuppression [11,12], the management of anticoagulation [13] and the center and surgeon’s experience [14].

The current risk indices for PTX are the pancreas donor risk index (PDRI) and the pre-procurement pancreas allocation suitability score (P-PASS) [15]. In 2008, the P-PASS was introduced by the Eurotransplant Pancreas Advisory Committee [16]. In the P-PASS, the donor characteristics age, BMI, intensive care unit (ICU) stay, cardiac arrest, sodium, amylase or lipase, and vasopressor therapy were assessed and a pancreas transplant donor with fewer than 17 points was considered an appropriate donor [16]. In 2010, the PDRI was established in the US by retrospectively analyzing 9401 pancreas transplantations [17]. Donor characteristics such as age, sex, race, BMI, type of donation (donation after brain death (DBD) or donation after circulatory death (DCD)), cold ischemia time (CIT) and type of transplant (SPK, PTA or PAK) were included in the risk index for 1-year pancreas graft survival [17]. Besides these scores, further composite risk models have been evaluated for PTX [15]. Although, different scores provide evidence in predicting pancreas graft survival, various studies could not confirm these findings [15]. The discrepancy in the prognostic value of the current scoring systems underlines the urgent need for a risk model for pancreas acceptance. The aim of the present study is to work out a composite donor risk model with a special focus on a particular cut-off value for pancreas acceptance.

## 2. Materials and Methods

### 2.1. Patient Selection and Data Acquisition

In this single-center study, we retrospectively analyzed the data of 97 patients undergoing PTX with SPK, PAK or PTA between 2010 and 2021 at the transplant center of Charité Campus Virchow Klinikum in Berlin. All patients transplanted during this period were included; the sole exclusion criterion was an age below 18 years. The follow-up period was up to 12 years after PTX. All patients fulfilled the Eurotransplant (ET) eligibility criteria. For data acquisition, our electronic patient database and ET Network Information System were used. The study was conducted in accordance with the Declaration of Helsinki. The retrospective data analysis was approved by the Institutional Ethics Committee of Charité (EA4/202/19).

### 2.2. Definitions

Early graft loss (EGL) was defined as graft pancreatectomy during the first 3 months after transplantation. Graft pancreatitis was defined as a value higher than three times the maximum reference value of serum lipase (60 U/L) after transplantation [18]. Acute rejection was defined as the need for immunosuppressive therapeutic intervention when rejection was highly suspected, since in most cases, no biopsy was performed to confirm the diagnosis. Graft thrombosis was detected CT-graphically or intraoperatively. Surgical complications observed during the first 30 days post-transplantation were evaluated according to the Clavien–Dindo classification [19].

### 2.3. Operative and Perioperative Standards and Surgeon’s Experience

As graft thrombosis is one of the main causes of EGL, anticoagulation regimens are an important factor in the early postoperative period. Our standard anticoagulation regimen starts intraoperatively with low-dose heparin (200 IU/h) at the time of reperfusion and is increased to 400 IU/h upon arrival at the ICU. Depending on kidney function, we switch to low-weight molecular heparin (LWMH) on postoperative day (POD) 4. Low-dose acetylsalicylic acid is added on POD 1.

Standard immunosuppression consisted of the intraoperative application of anti-thymocyte globulin (ATG; 1.5 mg/kg/BW), repeated to a cumulative dose of 7.5 mg/kg/BW. The time of ATG application depended on lymphocyte count [20]. Our maintenance immunosuppression is a triple therapy with calcineurin inhibitors (CNI), antimetabolites and steroids.

In our patient collective, all pancreas transplants were performed by highly experienced transplant and retrieval surgeons who also independently perform liver and kidney transplantations.

### 2.4. Statistical Analysis

All tables and figures were produced and statistical calculations performed using R version 4.2.1 an R Studio version 2022.07.2 for Windows (R Foundation for Statistical Computing, Vienna, Austria). The required packages gtsummary, tidyverse, survival and survminer were used. The data are presented as medians and interquartile ranges. Univariate analysis and Kaplan–Meier survival analysis with the log-rank test were performed to determine differences in graft survival. The multivariate Cox regression model with a hazard ratio (HR) was performed for the evaluation of the effect strength. A *p*-value of <0.05 was considered statistically significant.

## 3. Results

### 3.1. Recipient, Donor and Transplant Characteristics

Among 97 consecutive PTXs, 82 (85%) were performed with SPK, 11 (11%) with PAK and 4 (4%) with PTA. Thirteen (13%) patients received re-transplantations. The most common reason for PTX was type 1 diabetes (98%). The median recipient and donor ages were 43 and 33 years, respectively. The female-to-male ratio was 40% to 60% for recipients and 53% to 47% for donors. The recipient, donor and transplant characteristics are given in Table 1. Additional characteristics are displayed as supplementary material (Table S1).

### 3.2. Perioperative Outcome and Morbidity

The median follow-up after PTX was 5.2 (IQR 3.9, 7.8) years. The 1-year graft survival rate was 80%. The median ICU and hospital stay were 7 and 25 days, respectively. Forty-one patients (42%) developed major complications during their hospital stay, defined as grade 3b and higher according to the Clavien–Dindo classification. A total of 13 (13%) of the 97 patients developed complete graft thrombosis, 59 patients (61%) had graft pancreatitis, and 27 patients (28%) developed an acute rejection episode within 3 months after surgery. Further outcome data are summarized in Table 2.

### 3.3. Early Graft Loss (EGL)

EGL is defined as graft loss within 3 months after transplantation and occurred in 15 out of the 97 patients (15%).

#### 3.3.1. Donor Characteristics in EGL

In total, 20% of donors in the EGL group were male. The median donor age was 44 years and the median donor BMI was 22 kg/m^2^ in EGL recipients compared to 34 years (*p*-value 0.8) and 23 kg/m^2^ (*p*-value > 0.9) in the non-EGL group. The predominant cause of death in the EGL group was cerebrovascular accident (CVA), with 67% (n = 10), and 4 donors (27%) underwent mechanical cardiopulmonary resuscitation (mCPR). The mCPR rate in the non-EGL group was 16% (n = 13, *p*-value 0.3), and the CVA rate was 54% (n = 44, *p*-value 0.4)). The median donor lipase was 26 U/L, with a minimum value of 8 U/L and a maximum value of 193 U/L (IQR 20, 45) in the EGL group compared to 20 U/l with a minimum of 3 U/L and a maximum of 126 U/L (IQR 14, 34) in the non-EGL group (*p*-value 0.14). The median PDRI (*p*-value 0.5) and P-PASS (*p*-value 0.4) were similarly low in both groups. In patients with EGL, the median CIT was 602 (IQR 486, 658) min compared to 548 (IQR 427, 625) min (*p*-value 0.2) in non-EGL patients.

#### 3.3.2. Recipient Characteristics in EGL

The recipient characteristics were largely equally distributed. The recipient sex ratio (female to male) was 39% to 61% in non-EGL patients and 47% to 53% in EGL patients (*p*-value 0.6). The median recipient ages were 43 and 44 years (*p*-value 0.6), and the median BMI values were 24 and 23 kg/m^2^ (*p*-value 0.4), respectively. The percentages of patients with hypertension and vascular disease were 89% and 37% in non-EGL patients and 87% (*p*-value 0.7) and 40% (*p*-value 0.8) in patients with EGL.

Among the 15 patients who experienced EGL, graft thrombosis was detected in 11 patients (73%), and 4 patients lost their graft due to pancreatitis alone with patent vessels, whereas a further 5 patients had a combination of pancreatitis and thrombosis. Further characteristics and outcome data stratified by EGL are given in Table 3 and Table S2.

The graft survival between the EGL group and the non-EGL group is compared in the Kaplan–Meier curves in Figure S1 (*p* < 0.0001).

### 3.4. Potential Risk Factors for EGL

Before focusing on donor-related risk factors, we had to exclude potential peri- and postoperative risk factors for EGL. Anticoagulation therapy, measured as ptt on POD1, was at a median of 42 sec in the non-EGL group and 44 sec in the EGL group, respectively. Immunosuppression followed a standardized protocol with few exceptions. The operative volume of transplant surgeons was more heterogenous, but none of these factors were proven to be significant factors in the development of EGL, as shown by Chi-squared analysis (data shown in Table S2).

Donor age > 45 years, donor BMI > 25 kg/m^2^, donor lipase > 60 U/L, CVA as the cause of donor death, mCPR, CIT > 600 min and organ retrieval performed by another center as known risk factors for graft thrombosis and/or graft pancreatitis were analyzed as potential risk factors for EGL. However, none of these risk factors showed statistical significance in the univariate analyses. However, age > 45 years (*p* = 0.072) and BMI > 25 kg/m^2^ (*p* = 0.053) were close to the significance level. In the multivariate analysis via Cox regression, mCPR was the only risk factor that showed statistical significance, with a hazard ratio of 5.1 (CI 2.1–12.8). The other factors did not show statistical significance on multivariate analysis. Donor BMI > 25 kg/m^2^ showed a hazard ratio of 2.0 (CI 0.7–5.6). The hazard ratio of donor lipase > 60 U/L was 1.2 (CI 0.4–3.8), of donor age > 45 years was 1.8 (CI 0.4–7.9), and of CIT > 600 min was 1.6 (CI 0.7–3.9). The univariate and multivariate analyses of separate risk factors are summarized in Table 4 and Table 5.

### 3.5. Composite Risk Model

Based on the known potential risk factors, a composite risk model comprising a combination of risk factors for EGL was evaluated. The included donor characteristics were age > 45 years, BMI > 25 kg/m^2^, lipase > 60 U/L, CVA as the cause of death, mCPR, CIT > 600 min and organ retrieval not carried out by the recipient center. None of the patients in the EGL group had zero risk factors, only two (13%) had one risk factor and two (13%) had a combination of two risk factors. A total of 53% of the EGL group had three risk factors, compared to 28% in the non-EGL group. A total of 13% had a combination of four characteristics, versus 4.9% in the non-EGL group. A combination of five risk factors only occurred in the EGL group.

The combination of two or more risk factors was identified as a significant cut-off value for a higher risk for EGL, with a *p*-value of 0.046. The combination of three or more risk factors even showed a *p*-value of 0.003. Table 6 summarizes the composite risk model for EGL.

**Figure 1:**
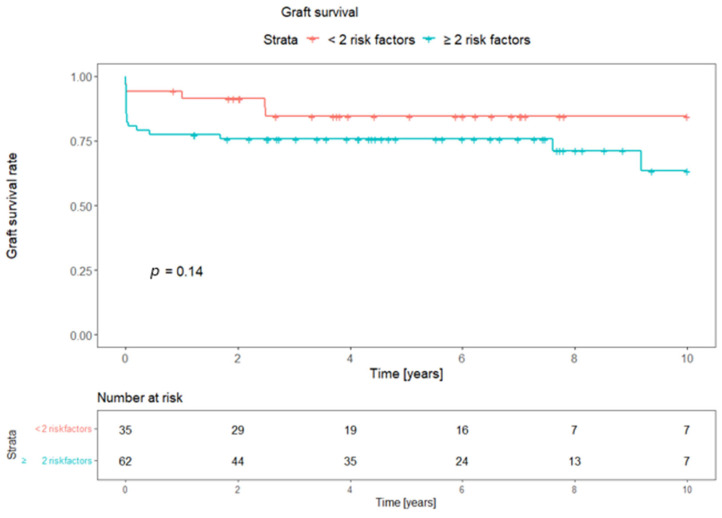
Kaplan–Meier curves depicting pancreas graft survival in the combination of two or more risk factors. *p*-value: log-rank comparison of survival curves.

**Figure 2:**
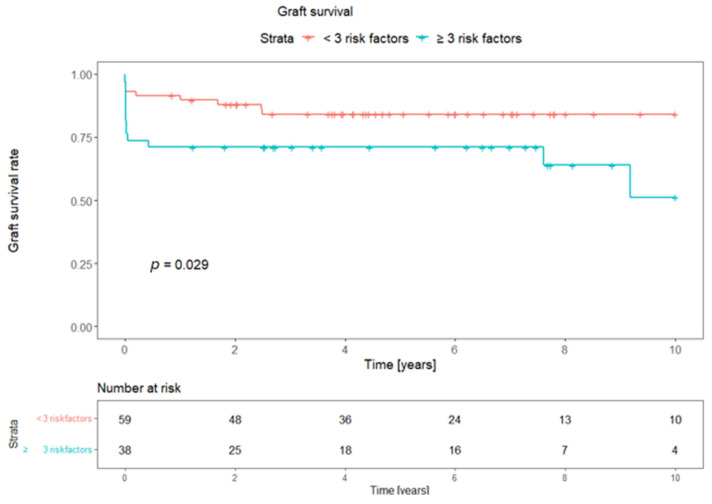
Kaplan–Meier curves depicting pancreas graft survival in the combination of three or more risk factors. *p*-value: log-rank comparison of survival curves.

The composite risk model was further evaluated for long-term graft survival. Pancreas graft survival was compared between less than two risk factors and a combination of two or more risk factors, showing 0.14 as the *p*-value of the log-rank comparison of the survival curves. The combination of three or more risk factors was identified as the cut-off value for a significant prediction of graft survival, with a *p*-value of 0.029, in the comparison of the Kaplan–Meier curves. Figure 1 and Figure 2 depict the graft survival curves and the risk tables.

## 4. Discussion

SPK and PTA can be life-extending and improve QOL for type-1 diabetics [5]. Notably, PTX has the highest incidence of non-immunologic failure of all solid-organ transplants [21]. EGLs are still a major reason for the loss of pancreas transplants [7]. The main causes of EGL are thrombosis, graft necrosis and pancreatitis [7,8,21,22]. It is hard to ultimately distinguish these complications from one another [18]. Acute pancreatitis can be generally divided into interstitial edematous pancreatitis and necrotizing pancreatitis [18]. In 5–10% of transplants, necrosis of the pancreatic parenchyma, the peri-pancreatic tissue or both occurs [18]. Early pancreas allograft thrombosis occurs in about 10% of pancreas transplants and usually leads to graft loss [21].

These outcome parameters depend on a multitude of pre-, peri- and postoperative factors. Center and surgeon experience, postoperative immunosuppression and anticoagulation may play pivotal roles.

Our applied mode of anticoagulation is in accordance with a recent meta-analysis on the topic by Li et. al. that clearly shows a two-fold lower risk of graft thrombosis and loss with prophylactic heparinization [23], although no precise protocol can be recommended due to a lack of evidence [5].

As immunosuppression in pancreas transplantation is not completely standardized, most studies report on a t-cell-depleting induction and maintenance therapy consisting of CNI, antimetabolite and steroids [24], as is standard in our center.

As center volume is shown to reduce morbidity and mortality in certain oncological procedures [25,26], as well as outcomes in kidney transplantation [27], consequently, low center volume could be shown to be a risk factor for graft failure in pancreas transplantation [14].

As we are faced with decreasing numbers in donors and transplantations [28], standardized training [29], fellowship programs and a reduction in the number of transplant centers may help to specialize surgeons in this field.

In order to legitimately focus on the pre-operative parameters of the donor and graft, we could successfully rule out significant differences in these three areas between the EGL and non-EGL group in our collective.

Therefore, our option to counteract EGL is the targeted individual selection of donor organs. Several risk scores are established to predict the suitability of organ donors for pancreas donation. Despite the use of pancreas donor risk indices, and in light of the shortage of organ donors, EGL and the complications thereof represent a significant problem. Because organ shortage is such a major problem, certain risk factors can be neglected in individual donor selection. One example of this is status post cardiac arrest and cardiopulmonary resuscitation. A statistical analysis of 13095 SPK transplants including 810 donors after resuscitation by Messner et al. demonstrated comparable death-censored pancreas graft survival [30].

In our retrospective single-center study, we found significantly reduced pancreas graft survival when the organs transplanted had three or more of the defined risk factors. The main cause was a dramatically higher rate of EGL. The factors that were defined as donor-related risk factors in our cohort of 97 pancreas transplants were age > 45 years, BMI > 25 kg/m^2^, lipase > 60 U/L, CVA as the cause of death, mCPR, CIT > 600 min and retrieval surgery by another center (Table 4). Other potential donor risk factors described in the literature are an elevated serum creatinine level, elevated serum sodium, dialysis, duration of ICU stay and the use of inotropes [15]. The fact that the use of these factors does not make sense to apply to our cohort is due to the previous selection; for example, no donor had a sodium level over a 170 mmol/L.

In our cohort of 97 PTX, we faced the challenge of identifying the risk factors for EGL, as the organs were already subject to preselection. Only 7 donors (7%) had an age above 45 years, only 15 donors (15%) had a BMI above 25 kg/m^2^, and a CIT > 600 min occurred in 34 cases (35%). Commonly used risk stratifications such as the PDRI [17] and P-PASS [16] were already in use for organ selection in our center. Also, the other described risk models are only of very limited use in a clinical setting, due to the very precise pre-selection of donors and organs [15]. As an example, the Minnesotan research group of Finger et al. published a composite risk model for technical failure in pancreas transplants combining the risk factors donor creatinine ≥ 2.5 mg/dl, donor age > 50 years, BMI ≥ 30 kg/m^2^ and cold preservation time > 20 h in the *American Journal of Transplantation* in 2013 [31]. Only four of our organs came from a donor with one of these characteristics, so this score cannot be applied to identify patients at risk for EGL in our center.

The challenge was to identify the risk profile in an already pre-selected pool of grafts.

Thus, the aim of our study was to identify useful cut-off values of the number of known risk factors for organ criteria to facilitate the decision of organ acceptance.

Donor age is a known risk factor for short- and long-term allograft loss in PTX. Its influence on complication rate and organ survival is proven in a multitude of studies [32,33,34,35,36,37,38]. Whereas older donors are accepted for kidney or liver transplantation, for PTX, donor age remains a critical exclusion criterion [10], excluding most of the DBD and DCD donors in an aging donor population. Several studies, such as the one by Krieger et al., who performed 91 pancreas transplants with donors over 45 years old [39], and our published data on long-term pancreas graft survival [3] established 45 years as the cut-off age that serves as an independent risk factor in PTX. Organs from donors above this cut-off age were also chosen as a risk factor in our collective. However, we could not show donor age to be a significant risk factor in its own right, which is in line with the results of the EXPAND multicenter study, which recommends an individual donor risk assessment for patients aged above 45 years [10,40].

Donor BMI is another risk factor described in multiple studies [33,34,35,36,41,42]. The single cut-off point of 30 kg/m^2^ did not show significantly reduced graft survival in the 2017 study by Alhamad et al., in which 9916 SPK transplants were evaluated [42]. Due to our rigorous pre-selection of donors, we chose 25 kg/m^2^ as the cut-off point. In our univariate analysis, a BMI over 25 kg/m^2^ was closest to being a significant risk factor for EGL, with a *p*-value of 0.053.

Donor lipase is described as predisposing factor and is also included in P-PASS [16]. Wullstein et al. investigated the difference between accepted and refused PTX for 1360 offered grafts and revealed elevated serum lipase of the donor as a frequent exclusion criterion in Europe [43]. We used donor lipase > 60 U/L as a risk factor and showed that it alone is not a risk driver for EGL or long-term graft survival, but a risk factor in combination with other predisposing criteria. This is consistent with the lack of the use of serum lipase as a single criterion in the literature [3,5], and that it is actually used as a combined criterion in P-PASS [16].

P-PASS also considers cardiac arrest, as we did [16]. In their article published in the *World Journal of Transplantation* in 2020, Munoz-Bellvis et al. analyzed risk criteria for donors in the literature, one of which is cardiac arrest [10]. They describe that hemodynamic instability contributes to inadequate organ perfusion and the development of thrombosis and pancreatitis. The authors therefore recommend a differentiated individual decision as to whether a donor organ should be used after cardiac arrest. In our multivariate analysis of graft survival via Cox regression, donor mCPR was the only statistically significant risk factor. Similarly to the conclusions of Munoz-Bellvis et al. [10] and Messner et al. [30], we do not suggest inevitable rejection of the organs but an individual decision based on further criteria.

Donor cause of death, in particular, CVA, is included in risk indices, such as in the PDRI [17]. In a retrospective multicenter study including 48,301 PTXs, Gruessner et al. describe the association between CVA as the cause of death and higher rates of graft loss [44]. Furthermore, there are data showing an association between CVA and allograft thrombosis [2,45]. In our cohort, CVA is more common in the EGL group than in the non-EGL group. Nevertheless, this result is not significant and CVA acquires its significance in the combination of factors. CVA is also the most common cause of death in our donor population, as only DBD grafts are available in Germany.

In 2012, our research group published a paper in the *Annals of Surgery* stating that prolonged CIT is significant risk factor for reduced graft survival [3]. CIT is also a factor considered in the PDRI [17] or the composite risk model of Finger et al. [31], and it is associated with graft survival in multiple studies [38,46,47]. Kasiske et al. demonstrated CIT to be the only independent risk factor for pancreas graft failure when the PDRI is included in the model [48]. The cut-off values vary in the literature: In our study from 2012, we were able to find a cut-off value of 14 h [3]; in their study from 2018, Sanchez et al. determined a value of 12 h [38]. Based on our collective, a value of 10 h proved to be reasonable for us. In the univariate analysis, we were able to show that CIT over 10 h was more common in the EGL group (53% vs. 32%), although this was not significant in our study sample.

Based on the fact that individual risk factors should not be an exclusion criterion and despite the fact that considerations of known risk factors were made during organ acceptance, we decided to evaluate a composite risk model and found the following: The combination of two or more risk factors was identified, with a *p*-value of 0.046, as a significant cut-off value for a higher risk of EGL, and the combination of three or more risk factors showed a *p*-value of 0.003. The combination of three or more risk factors was determined as the cut-off value for a significant prediction of graft survival, with a *p*-value of 0.029, in the comparison of the Kaplan–Meier curves. Our risk model can be a basis for further studies in larger cohorts.

We are aware that our study has limitations. First, this is an uncontrolled, retrospective and single-center study design. Second, we included a heterogeneous study population containing SPK, PAK and PTA, as well as first transplantations and re-transplantations. Third, only DBD grafts were included, as DCD is not an option in Germany. Fourth, our results could be influenced by the limited number of cases, a problem many studies in the field of pancreas transplantation are faced with. Despite these limitations, we found a significant difference in EGL and long-term pancreas graft survival. In principle, the consideration of a pancreas transplant in the case of three or more risk factors is not completely ruled out, and the decision must always be viewed individually and in the context of the donor–recipient properties. Nevertheless, this method, if commonly used by surgeons and endocrinologists, could significantly reduce the number of EGLs and subsequent graft losses and improve early allograft outcomes.

## 5. Conclusions

Despite diligent pre-selection, using various scores, the results of PTX leave a lot to be desired compared to other solid-organ transplantations. We could not demonstrate a significant influence of any single risk factor, possibly due to the limited number of patients and pre-selection in our collective. Still, we could demonstrate that an accumulation of the identified risk factors increases the risk of EGL. Consequently, we do not recommend dismissing pancreas grafts for any one single risk factor alone. Based on our results, we rather recommend adding up the single risk factors and avoiding transplanting grafts from donors with three or more of the described factors. In the presence of one or two risk factors, a careful evaluation of the donor and recipient characteristics should be considered in the process of acceptance decisions. As low numbers make large randomized controlled trials an unrealistic prospect in the field of pancreas transplantation, we summarize that a multicenter analysis with a larger patient cohort is warranted to confirm the generalizability and applicability of our results.

## Figures and Tables

**Table 1:** Recipient, donor and transplant characteristics.

Recipient Characteristics	n = 97 ^1^
Diagnosis	
Type 1 diabetes	95 (98%)
Type 2 diabetes	1 (1%)
Pancreatitis	1 (1%)
Recipient sex ratio (F:M)	39 (40%):58 (60%)
Recipient age (y)	43 (36, 50)
**Donor Characteristics**	
Donor sex ratio (F:M)	51 (53%):46 (47%)
Donor age (y)	33 (22, 42)
Donor BMI (kg/m^2^)	23 (21, 25)
Donor cause of death	
CVA	54 (56%)
Trauma	31 (32%)
Anoxia	8 (8%)
Others	4 (4%)
Donor mCPR	17 (18%)
Donor lipase (U/L)	21 (14, 36)
PDRI	1.06 (0.85, 1.28)
P-PASS	17 (15, 18)
**Transplant Characteristics**	
Transplant type	
SPK	82 (85%)
PAK	11 (11%)
PTA	4 (4%)
Re-transplantation	13 (13%)
Retrieval by own center	40 (41%)
Time of perfusion (min)	68 (60, 78)
Cold ischemia time (min)	555 (463, 646)
Anastomosis time (min)	26 (23, 32)

^1^ n (%); median (IQR). BMI, body mass index; CVA, cerebrovascular accident; d, days; mCPR, mechanical cardiopulmonary resuscitation; PAK, pancreas transplanted after kidney; PDRI, pancreas donor risk index; P-PASS, pre-procurement pancreas suitability score; PTA, pancreas transplanted alone; y, years; SPK, simultaneous pancreas and kidney.

**Table 2:** Outcome data.

Outcome	n = 97 ^1^
Post-transplant follow-up (y)	5.2 (3.0, 7.8)
Pancreas graft survival (y)	4.4 (2.0, 7.7)
1-year graft survival	80 (82%)
Graft loss	22 (23%)
Early graft loss	15 (15%)
Pancreatectomy	17 (18%)
Hospital stay (d)	25 (19, 44)
ICU stay (d)	7 (5, 12)
Clavien–Dindo	
3a	16 (28%)
3b	37 (65%)
4b	2 (3.5%)
5	2 (3.5%)
Graft pancreatitis	59 (61%)
Graft thrombosis	28 (29%)
Complete graft thrombosis	13 (13%)
Acute rejection	27 (28%)

^1^ n (%); median (IQR). d, days; ICU, intensive care unit; y, years.

**Table 3:** Characteristics and outcomes stratified by early graft loss.

Characteristic	Graft Survival > 3 Months n = 82 ^1^	Early Graft Loss n = 15 ^1^	*p*-Value ^2^
**Recipient Characteristics**			
Transplant type			
PAK	8 (9.8%)	3 (20%)	
PTA	3 (3.7%)	1 (6.7%)	
SPK	71 (87%)	11 (73%)	
Re-transplantation	10 (12%)	3 (20%)	0.4
**Donor Characteristics**			
Donor sex ratio (F:M)	39 (48%):43 (52%)	12 (80%):3 (20%)	0.021
Donor age (y)	34 (22, 42)	33 (22, 42)	0.8
Donor BMI (kg/m^2^)	23 (21, 25)	22 (21, 26)	>0.9
Donor cause of death			
CVA	44 (54%)	10 (67%)	0.4
Trauma	27 (33%)	4 (27%)	0.8
Anoxia	8 (9.8%)	0 (0%)	0.4
Others	3 (3.7%)	1 (6.7%)	0.5
Donor mCPR	13 (16%)	4 (27%)	0.3
Donor lipase (U/L)	20 (14, 34)	26 (20, 45)	0.14
PDRI	1.05 (0.86, 1.28)	1.17 (0.81, 1.36)	0.5
P-PASS	17 (15, 18)	16.5 (15, 17)	0.4
**Transplant Characteristics**			
Retrieval by own center	35 (43%)	5 (33%)	0.5
Time of perfusion (min)	68 (58, 78)	68 (66, 75)	0.5
Cold ischemia time (min)	548 (427, 625)	602 (486, 658)	0.2
Anastomosis time (min)	26 (23, 31)	28 (24, 35)	0.4
**Outcome**			
Graft pancreatitis	50 (61%)	9 (60%)	>0.9
Graft thrombosis	16 (20%)	12 (80%)	<0.001
Complete graft thrombosis	2 (2.4%)	11 (73%)	<0.001

^1^ n (%); median (IQR). ^2^ Fisher’s exact test; Pearson’s Chi-squared test; Wilcoxon rank sum test. BMI, body mass index; CVA, cerebrovascular accident; d, days; mCPR, mechanical cardiopulmonary resuscitation; PAK, pancreas transplanted after kidney; PDRI, pancreas donor risk index; P-PASS, pre-procurement pancreas suitability score; PTA, pancreas transplanted alone; SPK, simultaneous pancreas and kidney; y, years.

**Table 4:** Risk factors for early graft loss.

Characteristic	Graft Survival > 3 Months n = 82 ^1^	Early Graft Loss n = 15 ^1^	*p*-Value ^2^
Donor age > 45 (y)	4 (4.9%)	3 (20%)	0.072
Donor BMI > 25 (kg/m^2^)	10 (12%)	5 (33%)	0.053
Donor lipase > 60 (U/L)	11 (14%)	3 (21%)	0.4
Donor cause of death: CVA	44 (54%)	10 (67%)	0.4
Donor mCPR	13 (16%)	4 (27%)	0.3
Cold ischemia time > 600 (min)	26 (32%)	8 (53%)	0.11
Organ retrieval by another center	47 (57%)	10 (67%)	0.5

^1^ n (%). ^2^ Fisher’s exact test; Pearson’s Chi-squared test. BMI, body mass index; CVA, cerebrovascular accident; mCPR, mechanical cardiopulmonary resuscitation.

**Table 5:** Multivariate analysis of risk factors.

Risk Factor	*p*-Value	HR	95% CI of HR
Donor age > 45 (y)	0.437	1.799	0.410–9.899
Donor BMI > 25 (kg/m^2^)	0.188	2.003	0.712–5.640
Donor lipase > 60 (U/L)	0.720	1.228	0.400–3.774
Donor mCPR	<0.0001	5.149	2.071–12.805
CIT > 600 (min)	0.291	1.614	0.664–3.928

Log-rank test, likelihood ratio test. BMI, body mass index; CI, confidence interval; CIT, cold ischemia time; HR, hazard ratio; mCPR, mechanical cardiopulmonary resuscitation.

**Table 6:** Composite risk factors for early graft loss.

Characteristic	Graft Survival > 3 Months n = 82 ^1^	Early Graft Loss n = 15 ^1^	*p*-Value ^2^
Number of risk factors			
0	7 (8.5%)	0 (0%)	0.6
1	26 (32%)	2 (13%)	0.2
2	22 (27%)	2 (13%)	0.3
3	23 (28%)	8 (53%)	0.072
4	4 (4.9%)	2 (13%)	0.2
5	0 (0%)	1 (6.7%)	0.2
Combination of two risk factors			0.046 *
<2 risk factors	33 (40%)	2 (13%)	
≥2 risk factors	49 (60%)	13 (87%)	
Combination of three risk factors			0.003 *
<3 risk factors	55 (67%)	4 (27%)	
≥3 risk factors	27 (33%)	11 (73%)	

^1^ n (%). ^2^ Fisher’s exact test; Pearson’s Chi-squared test. * *p*-Value < 0.05 represents significant results.

## Data Availability

The data presented in this study are available on request from the corresponding author. The data are not publicly available due to ethical restrictions.

## Supplementary Materials

The following supporting information can be downloaded at: https://www.mdpi.com/article/10.3390/jcm13175177/s1, Table S1. Additional Recipient, Donor and Transplant Characteristics. Table S2. Additional Characteristics and outcomes stratified by early graft loss. Figure S1. Kaplan-Meier curves depicting pancreas graft survival for early graft loss.
