# Supplementary material for: Outcome-Orientated Organ Allocation—A Composite Risk Model for Pancreas Graft Evaluation and Acceptance
Source: J Clin Med. 2024 Aug 31;13(17):5177. doi: 10.3390/jcm13175177 (PMC11396207; doi:10.3390/jcm13175177)
Supplement: Supplementary file 1 [file jcm-13-05177-s001.zip › jcm-3103643-supplementary.pdf]

Journal of
Clinical Medicine

MDPI

Table S1. Additional Recipient, Donor and Transplant Characteristics.

**Recipient Characteristics**	**n = 97**[1]
Recipient BMI (kg/m^2)	24 (22, 25)
Recipient hypertension	86 (89%)
Recipient vascular disease	36 (37%)
Dialysis use preemptive	79 (81%)
**Donor Characteristics**	
Donor hypertension	13 (13%)
Donor ICU stay (d)	2 (2, 4)
Donor creatinine (mg/dl)	0.70 (0.59, 0.90)
Donor sodium (mmol/l)	147 (142, 152)

[1] n (%); Median (IQR)

BMI, body mass index; d, days; ICU, intensive care unit.

Table S2. Additional Characteristics and outcomes stratified by early graft loss.

Characteristics	Graft survival > 3 month N = 82[1]	Early graft loss N = 15[1]	p-value[2]
**Recipient Characteristics**			
Recipient sex ratio (F:M)	32 (39%) : 50 (61%)	7 (47%) : 8 (53%)	0.6
Recipient age (y)	43 (36, 50)	44 (32, 48)	0.6
Recipient BMI (kg/m^2)	24 (22, 25)	23 (22, 25)	0.4
Recipient hypertension	73 (89%)	13 (87%)	0.7
Recipient vascular disease	30 (37%)	6 (40%)	0.8
**Donor Characteristics**			
Donor hypertension	10 (12%)	3 (20%)	0.4
Donor ICU stay (d)	3 (2, 4)	2 (1, 2)	0.031
Donor creatinine (mg/dl)	0.70 (0.60, 0.90)	0.66 (0.49, 0.83)	0.3
Donor sodium (mmol/l)	148 (142, 152)	144 (142, 150)	0.5
**Transplant Characteristics**			
PTT POD1 (sec)	42 (37, 51)	44 (38, 51)	0.7
Induction treatment			0.11
ATG	*79 (98%)*	*13 (87%)*	
Basiliximab	*2 (2.5%)*	*1 (6.7%)*	
ATG, Plasmapheresis, Infliximab	*0 (0%)*	*1 (6.7%)*	
Surgeons volume in our cohort			0.2
> 15 transplants	*49 (60%)*	*6 (40%)*	
≤ 15 transplants	*33 (40%)*	*9 (60%)*	

[1] n (%); Median (IQR)

[2]Fisher's exact test; Pearson's Chi-squared test; Wilcoxon rank sum test

ATG, anti-thymocyte globulin; BMI, body mass index; d, days; ICU, intensive care unit; POD, post-operative day; PTT, partial thromboplastin time; y, years.

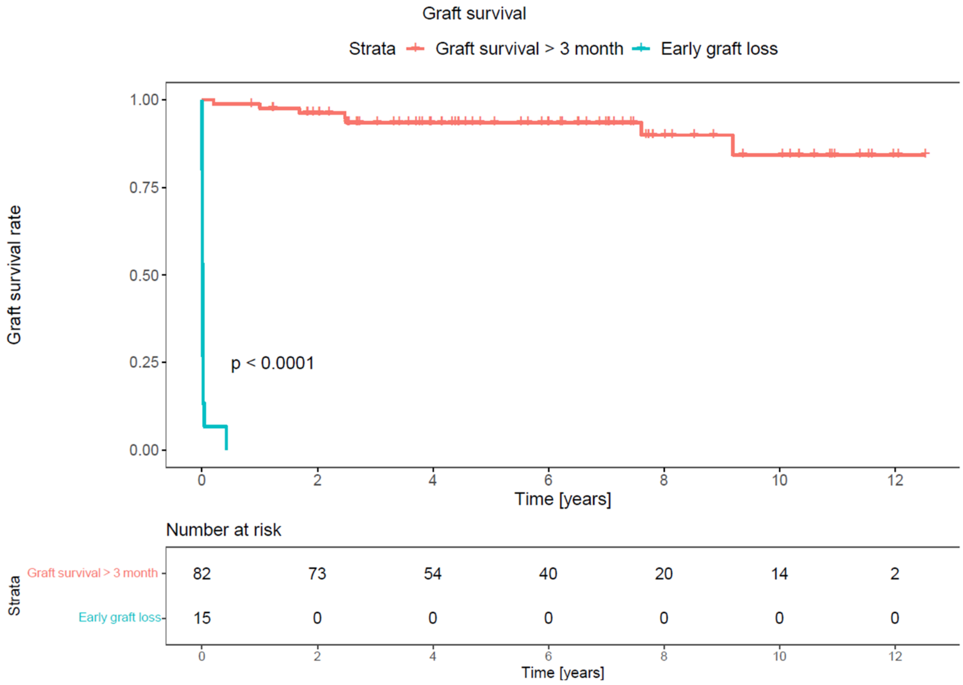

Figure S1. Kaplan-Meier curves depicting pancreas graft survival for early graft loss. p-value: log-rank comparison of survival curves.
